# The association between the admission to wards with open- vs. closed-door policy and the use of coercive measures

**DOI:** 10.3389/fpsyt.2023.1268727

**Published:** 2023-10-25

**Authors:** Jana S. Krückl, Julian Moeller, Lukas Imfeld, Sabine Schädelin, Lisa Hochstrasser, Roselind Lieb, Undine E. Lang, Christian G. Huber

**Affiliations:** ^1^University Psychiatric Clinics Basel (UPK), University of Basel, Basel, Switzerland; ^2^Division of Clinical Psychology and Epidemiology, Department of Psychology, University of Basel, Basel, Switzerland; ^3^Department Clinical Research, c/o University Hospital Basel, Basel, Switzerland

**Keywords:** open-door policy, open doors, coercive measures, seclusion, psychiatry

## Abstract

**Introduction:**

Psychiatric treatment on a ward with open-door policy is associated with reduced numbers of coercive measures. The effect of the door policy of previous stays, however, has not been investigated.

**Methods:**

The data set consisted of 22,172 stays by adult inpatients in a psychiatric university hospital between 2010 and 2019. Pairs of consecutive stays were built. The outcome variable was the occurrence of coercive measures during the second stay.

**Results:**

Compared to treatments on wards with a closed-door policy at both stays, treatments on wards with an open-door policy at the second stay had smaller odds for coercive measures (OR ranging between 0.09 and 0.33, *p* < 0.01). In addition, coercive measures were more frequent in treatment histories where patients previously treated on a closed ward were admitted to a ward with an open-door policy and subsequently transferred to a ward with a closed-door policy at the second stay (OR=2.97, *p* = 0.046).

**Discussion:**

Treatment under open-door policy is associated with fewer coercive measures, even in patients with previous experience of closed-door settings. The group of patients who were admitted to a ward with an open-door, then transmitted to a ward with a closed-door policy seem to be prone to experience coercive measures. Clinical strategies to keep these patients in treatment in an open-door setting could further reduce coercive measures.

## 1. Introduction

Whether doors of psychiatric inpatient wards are kept open or closed, highly varies between countries, hospitals, and wards (1). Whereas psychiatric hospitals in German-speaking countries increasingly rely on open-door policies, hospitals in the United Kingdom are mostly keeping the doors of acute psychiatric wards closed (2, 3). While recent research findings have questioned the beneficial effect of closed wards in the treatment of psychiatric patients (4–6), some studies also reported detrimental effects. Patients (7) as well as staff (8) perceived wards with a closed-door policy as confinement and a non-caring environment. This may cause additional emotional problems in patients and hinder recovery (9). Ward crowding (10) and difficult social interactions between patients and staff predicted aggressive behavior in patients (11). Aggression, in turn, is associated with the indicated use of coercive measures. Several researchers found that the most frequently reported reason for coercive measures in psychiatric inpatient settings is aggressive behavior by the patient (12, 13).

Open-door policies – consisting of various interventions with the aim of a patient-centered and recovery-oriented care (14) – and their association with the use of coercive measures have been investigated by several researchers in the past years. Data from Swiss (5) and German psychiatric hospitals (6) showed less coercive measures, namely seclusion and forced medication, during psychiatric treatments on wards with an open-door policy compared to wards with a closed-door policy. Moreover, Schneeberger et al. (6) reported less aggressive incidents on wards with an open-door policy. However, other studies did not find consistent effects after the implementation of an open-door policy (15, 16). So far, previous admissions to wards with open-door vs. closed-door policy have not been accounted for when examining the link between door policies and the use of coercive measures. However, previous experience of coercion is known to be related to more coercion in the future (12, 17), and experiencing a less restrictive open-door setting with less coercion might in turn positively influence future treatment episodes.

The present paper aims to investigate whether the ward types during the most recent and during the current inpatient hospital stay are associated with the use of coercive measures during the current stay.

## 2. Materials and methods

### 2.1. Framework

The University Psychiatric Clinics (UPK) Basel, University of Basel, Switzerland, is a large psychiatric university hospital providing psychiatric in- and outpatient services for a population of approximately 202,000 people living in the city of Basel and the surrounding area. Since 2010, the hospital’s management aimed to implement a less restrictive policy in the UPK (5). In the Clinic for Adults and the Private Clinic, 233 beds on 15 wards were available for inpatient treatment on January 1, 2010. Nine of these wards followed an open-door policy and one a closed-door policy for the whole observation period, five wards were initially closed and implemented an open-door-policy at some point of the observation period [two wards in August 2011, one in December 2013, one in June 2014 and one in September]. Besides of the opening of ward doors, the hospital-wide approach of an open-door policy consisted of numerous additional strategies, e.g., increasing one-to-one care in crisis situations, training for de-escalation strategies, and standardization of crisis management for suicidality and aggression. Overall, the aim was and still is to implement an orientation of a patient-centered and recovery-oriented focus in the treatment of psychiatric patients. See here for a detailed description and evaluation of the open-door policy concepts as implemented in the UPK Basel (18–20).

### 2.2. Sample

The data set consisted of all inpatient stays by adult patients treated in the Clinic for Adults and the Private Clinic of the UPK Basel between January 1, 2010 and December 31, 2019, who had more than one hospital stay during the observation period. Patients were excluded if they were younger than 18 years old, or if they did not fulfill the time criteria for an inpatient treatment (length of stay <1 day). Moreover, patients whose inpatient treatment exceeded the observation period (i.e., who were admitted within the study period, but were discharged after December 31, 2019) were excluded. No further exclusion criteria were defined to ensure a naturalistic sample. In total, 17,054 follow-up stays by 5,118 inpatients were included, i.e., in total 22,172 stays (5,118 first stays +17,054 follow-up stays; 74.6% of all inpatient stays in the above-mentioned period, N = 29,733).

### 2.3. Documentation of clinical data and measures

Clinical and treatment data are continuously documented using Medfolio software (current version: 2.2.0.1.1455, Release 3.0.0.0; NEXUS AG, Villingen-Schwenningen, Germany). The data could be extracted on a daily basis to facilitate process management and enable statistical analysis.

For each stay, we extracted data on ward type (with open-door policy vs. with closed-door policy) as well as individual demographic and clinical data of the patient including age, gender, diagnoses, type of admission (voluntary vs. involuntary), and symptom severity at admission. This information was documented by the psychiatrist responsible for the respective patient. In Switzerland, coercive measures (seclusion, forced medication, and restraint) must be documented due to legal regulations. For the present study, two types of coercive measures were defined as the main outcome variables:

1. Seclusion: involuntary isolation with or without psychopharmacological treatment. Isolation is defined as the involuntary placement of a patient alone in a locked room.
2. Forced medication: involuntary intake of oral or the application of intramuscular medication without being secluded or restrained.

The third type of coercive measure – physical restraint (defined as mechanical restraint using belts or straps) – is not administered in the UPK Basel and was therefore not available for the current analyses. Involuntary hospitalization is an additional coercive measure. As only public health officers and local authorities are permitted to mandate an involuntary hospitalization in the canton of Basel-City, it is questionable if changes in the hospital policies impact these decisions. Involuntary hospitalization was thus not included as an outcome variable. However, based on recent research, it was included as a confounder. As aggressive behavior, involuntary admission and treatment on closed wards are linked (12, 21, 22), many incidents of coercion indeed occur on ward with a closed-door policy; however, by far not all as the hospital is not obligated to admit voluntary patients to an open and involuntary patients to a closed ward. On the contrary, over the course of implementing the open-door policy in our hospital, more and more involuntary patients were admitted to open wards. And the vast majority of involuntarily admitted patients do not experience any further coercive measures. As a consequence of this, coercive measures are applied on closed as well as on open wards. For further information concerning the definition of coercive measures, please consider the medical-ethical guidelines of the Swiss Academy of Medical Sciences [Swiss Academy of Medical Sciences (23)].

The authors assert that all procedures contributing to this work comply with the ethical standards of the relevant national and institutional committees on human experimentation and with the Helsinki Declaration of 1975, as revised in 2008. All procedures involving human subjects were approved by the Ethics Committee of Northwestern and Central Switzerland (EKNZ; Project-ID: 287–13 / PB_2020–00029). The study was categorized and accepted by the ethics committee as further use of routine data without consent according to HRA Art.34/HRO.

### 2.4. Statistical analyses

Frequencies and percentages are given for categorical data. For continuous data, the median as well as the 1st and the 3rd quartile are presented.

For each stay, we assessed whether the ward on which a patient was admitted followed an open-door or a closed-door policy during the time of their treatment. The ward type was operationalized using the following factors: open (O), open-closed (O/C; indicating that the patient was admitted on a ward with an open-door policy but had to be transferred to a ward following a closed-door policy at some point of the stay) and closed (C). The status “closed” was treated as an absorbing status. Even if a patient was transferred from a ward with a closed-door policy to a ward with an open-door policy during the stay, their stay was still considered as “closed.”

**Figure 1:**
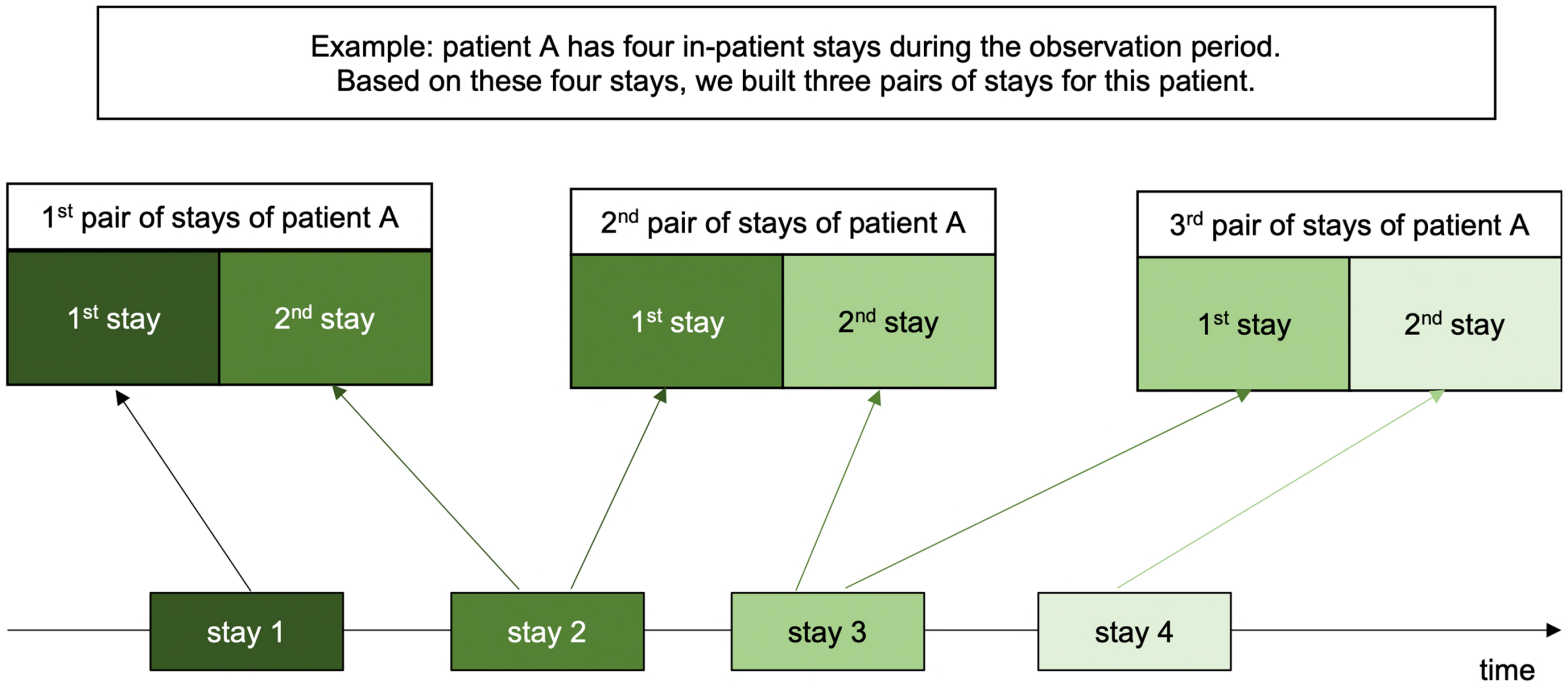
Procedure of building pairs of stays for each patient.

Using these three statuses, we built pairs of consecutive stays for each patient. In patients with three or more stays the pairs were constructed as follows: stay 1 + stay 2, stay 2 + stay 3, etc. (see Figure 1). This variable represents the recent treatment history as a combination of the ward types of two consecutive stays (“O – O,” “O – O/C,” “O – C,” “O/C – O,” “O/C – O/C,” “O/C – C,” “C – O,” “C – O/C” or “C – C”). The combinations “O/C – O/C” and “C – O/C” were, however, combined to one category due to small numbers (N = 12 and N = 94, respectively). The sequence of “C – C” was used as reference level.

The primary endpoint of the analyses is the presence of coercive measures at the second inpatient stay of each created pair (“no coercive measure” vs. “forced medication and/or seclusion”). This variable indicates whether one of the measures was taken at least once during the stay. The association between the combination of the ward types and the coercive measures is analyzed in a logistic model using the above-mentioned contrasts “no coercive measures” and “forced medication and/or seclusion.”

In past research, some variables have been shown to be associated with the use of coercive measures. These variables are included in the model as confounders, namely age (24), gender (24, 25), diagnoses (13, 24–26), symptom severity (13, 24, 25), involuntary admission (12, 17), previous experiences of coercive measures (17) and duration of stay (27). All confounders were assessed at the first stay of each pair of stays. To explore the relationships between predictors, the outcome variable as well as the confounders, we calculated zero-order correlations (Spearman’s correlation) based on the pairwise complete cases.

For the variable “previous coercive measures,” we coded two binary variables for coercive measures at the first stay of each pair of stays indicating if a patient experienced an episode of seclusion or forced medication at least once during their first stay of each pair of stays. Three items of the Health of the Nations Outcome Scales [HoNOS; (28)] were included as a proxy for symptom severity: item (1) overactive, aggressive, disruptive, or agitated behavior (called “aggressive behavior” in the following tables); item (2) non-accidental self-injury (“auto-aggressive behavior”), and item (6) problems with hallucinations and delusions (“psychotic symptoms”). They are rated on a scale from 0 to 4. For the present analyses, these ratings were coded in a binary variable of “not present” (HoNOS rating of 0–1) and “present” (HoNOS rating of 2–4). The HoNOS is recorded in the digital patient file as part of the standard admission procedure at the UPK Basel. Clinical diagnoses were assessed according to ICD, 10th revision (29). Every block of the chapter “Mental and Behavior Disorder (F00-F99)” is depicted in a binary variable, coded “yes” if the patient had a diagnosis in this block [e.g., a mood (affective) disorder] and “no” if not. Moreover, we included another binary variable indicating if any other ICD-10 diagnosis (besides the ones from the F-chapter) was present as well as a variable “number of secondary diagnoses” (including the whole ICD-10). In total, 21 confounding variables were included in the model.

Missing values were imputed using multiple imputations by chained equations according to van Buuren & Groothuis-Oudshoorn (30). All variables available were used for the imputations. Results are pooled using Rubin’s rule. A significance level of α = 5% (two-sided) was applied. Generalized estimating equations (GEE) was used to account for dependence in patients with three or more stays (and thus having more than one pair). No correction for multiple testing was performed because of the exploratory nature of the analyses. The odd ratios, 95%-confidence intervals and *p*-values are presented.

## 3. Results

Of the total of 5,118 inpatients, 43.0% had two inpatient stays during the observation period (N = 2,201), 20.6% had three inpatient stays (N = 1,054), 10.1% had four (N = 517), 7.3% had five (N = 376), and 12.4% had between 6 and 10 inpatient stays (N = 636). 334 inpatients of this sample (6.5%) had more than 11 stays during the observation period.

The results of the zero-order correlations are presented in the Supplementary Appendix Table. Two pairs of variables appeared to correlate fairly high: “psychotic symptoms at admission” and “F2 Schizophrenia, schizotypal and delusional disorders” (ρ = 0.59) as well as “number of secondary diagnoses” and “F1 Mental and behavioral disorders due to psychoactive substance use” (ρ = 0.64).

In Table 1, demographic and clinical characteristics of all stays (all patients, several stays per patient) are shown. The data refers to the first stay of each pair of stays.

**Table 1:** Demographic and clinical characteristics of the sample at their first stay (N = 17,054).

		Median [Q1, Q3] resp. N (%)	
Age (in years)		44.94	[33.87, 55.49]
Gender (female)		8,706	(51.0%)
Forced measures			
No measure		16,031	(94.0%)
Forced medication		43	(0.3%)
Seclusion		980	(5.7%)
Involuntary hospitalization		1774	(10.4%)
Aggressive behavior at admission		3,352	(31.0%)
Auto-aggressive behavior at admission		1,593	(14.8%)
Psychotic symptoms at admission		3,229	(30.5%)
Diagnoses (ICD-10)			
F0 Organic, including symptomatic, mental disorders		941	(5.5%)
F1 Mental and behavioral disorders due to psychoactive substance use		9,763	(57.2%)
F2 Schizophrenia, schizotypal and delusional disorders		4,622	(27.1%)
F3 Mood (affective) disorders		7,132	(41.8%)
F4 Neurotic, stress-related and somatoform disorders		3,271	(19.2%)
F5 Behavioral syndromes associated with physiological disturbances and physical factors		503	(2.9%)
F6 Disorders of adult personality and behavior		3,885	(22.8%)
F7 Mental retardation		390	(2.3%)
F8 Disorders of psychological development		113	(0.7%)
F9 Behavioral and emotional disorders with onset usually occ. in childhood and adolescence		686	(4.0%)
Any other non-psychiatric diagnosis		3,839	(22.5%)
Number of secondary diagnoses		2.00	[0.00, 3.00]
Length of stay (in days)		14.00	[5.00, 36.00]

Q1 corresponds to the 1st quartile, Q3 to the 3rd quartile. In case of presence of forced medication and seclusion, these stays are listed under the category “seclusion”.

Table 2 shows the frequencies of coercive measures at the second stay of each pair. These appear to be only slightly different to the frequency of the coercive measures at the first stay (see Table 2). At the second stay, no coercive measures were applied in most cases (N = 16,138, 94.6%). In 5.2% of the inpatients stays (N = 879) an episode of seclusion (with or without forced medication) occurred. Forced medication alone was rare (N = 37, 0.2%). Coercive measures were most frequent in treatment histories where the admission to the second stay was either on a ward with a closed-door policy (“O – C”: N = 81, 14.9%; “O/C – C”: N = 23, 23.5%; “C – C”: N = 335; 14.3%) or when first admitted to a ward with an open-door policy and subsequently transferred to a ward with a closed-door policy (“O – O/C“: N = 19, 18.6%; “C – O/C”: N = 36, 33.9%).

**Table 2:** Frequency of coercive measures during the second stay of each pair (outcome variable).

Sequence of ward types	Coercive measures						All
	No measures		Forced medication		Seclusion		
	*n*	(%)	*n*	(%)	*n*	(%)	
O – O	11,959	(97.0%)	18	(0.2%)	347	(2.8%)	12,324
O – O/C	83	(81.4%)	0	(0.0%)	19	(18.6%)	102
O – C	463	(85.1%)	3	(0.6%)	78	(14.3%)	544
O/C – O	145	(98.0%)	1	(0.7%)	2	(1.4%)	148
O/C – C	75	(76.5%)	1	(1.0%)	22	(22.5%)	98
C – O	1,339	(96.1%)	3	(0.2%)	51	(3.7%)	1,393
C – O/C	70	(66.0%)	1	(0.9%)	35	(33.0%)	106
C – C	2,004	(85.7%)	10	(0.4%)	325	(13.9%)	2,339
All	16,138	(94.6%)	37	(0.2%)	879	(5.2%)	17,054

In case of presence of forced medication and seclusion, these stays are listed under the category “seclusion”.

In Table 3, the main results are presented showing that specific sequences of ward types at the most recent and the current stay are stronger associated with the use of coercive measures during the current stay. The following combination of ward types was shown to have reduced odds for coercive measures during the current stay compared to patients who were treated on a ward with a closed-door policy at both stays: “O – O” (OR = 0.33, 95% CI [0.17, 0.63], *p* < 0.01), “O/C – O” (OR = 0.09, 95% CI [0.02, 0.44], *p* < 0.01), and “C – O” (OR = 0.26, 95% CI [0.11, 0.61], *p* < 0.01). These three combinations of ward types had three to 10 times smaller odds for experiencing coercive measures at their current stay. The treatment history of “C – O/C,” however, appeared to have almost three times higher odds for coercive measures during the current stay (OR = 2.97, 95% CI [1.02, 8.61], *p* = 0.046) compared to treatment on wards with a closed-door policy at both stays. These effects persisted even when all the above-mentioned confounders were included in the model.

**Table 3:** Association between the sequence of ward types at the most recent and the current stay with coercive measures during the current stay.

	*OR*	95% CI	*p*
(Intercept)	0.01	[0.00, 0.04]	<0.01
Treatment history			
O – O	0.33	[0.17, 0.63]	<0.01
O – O/C	2.50	[0.87, 7.18]	0.088
O – C	1.50	[0.65, 3.44]	0.342
O/C – O	0.09	[0.02, 0.44]	<0.01
O/C – C	1.41	[0.46, 4.37]	0.547
C – O	0.26	[0.11, 0.61]	<0.01
C – O/C	2.97	[1.02, 8.61]	0.046

As a reference level, the combination of ward types “C – C” was chosen. Also included in the model were 21 confounders (age, gender, forced medication and seclusion, involuntary hospitalization, the HoNOS items 1, 2 and 6, length of stay, type and number of diagnoses).

## 4. Discussion

We investigated the association between the sequence of ward types (open- vs. closed-door policy) with the use of coercive measures in a large psychiatric university hospital. Almost 95% of the inpatients did not experience any coercive measure during either of their stays. Seclusion with or without forced medication was considerably more frequent than forced medication alone. Compared to treatments on a ward with closed-door policy at both stays, treatments on a ward with open-door policy had substantially smaller odds for coercive measures during the current stay. The treatment on a ward with closed-door policy at the most recent stay and the transfer from a ward with an open-door to one with a closed-door policy at the current stay, however, had almost three times higher odds compared to the treatment on a ward with a closed-door policy at both stays. All these effects remained persistent when confounders that have been previously shown to be associated with the use of coercive measures were included.

Our results show that the sequence of ward types predicted coercive measures. Treatment on wards with an open-door policy had smaller odds for coercive measures at the current stay, even when patients had received treatment in a closed-door setting during their previous hospitalization. Overall, the door policy during the current stay seems to be more relevant when predicting coercive measures at the current stay (compared to the door policy during the most recent stay). These findings support previous research that showed that there are less coercive measures on open wards (5, 6, 31). The inclusion of numerous variables that are linked to the use of coercive measures strengthens these results.

However, due to the nature of our analyses, it is not possible to infer a causal link between the door-policy and coercive measures. The data source for this naturalistic observational study originated from routine clinical data. While this strengthens clinical validity, this means that there was no random allocation of patients to open-door or closed-door settings. Thus, patients who were at an increased risk for coercive measures from the admitting psychiatrist’s point of view might, in principle, have been more likely to be admitted to a closed-door setting for safety reasons. However, the psychiatric hospital enforces an open-door policy where patients are admitted to specific wards based on their psychiatric diagnosis and treatment continuity, and severe cases are distributed equally over all wards. Thus, this bias is actively minimized in the clinical setting in question. Furthermore, other factors could be potentially relevant for the occurrence of coercion. Addressing this point, we included numerous confounders that have been shown to be related to coercive measures. Certainly, due to the observational nature of this study, potential effects of other variables that have not been assessed (e.g., attitude toward coercive measures of the staff) may also have affected the odds of coercive measures. However, to our best knowledge, we included the most relevant confounders. Furthermore, the only strategy to draw causal conclusions from the door policy to coercive measures would be by conducting a randomized controlled trial. Yet, this research design is ethically problematic and difficult to realize as assigning suicidal and/or aggressive patients randomly to wards with either an open- vs. a closed-door policy may threaten the safety of the patient and/or others. However, in recent years, some researchers have followed promising study designs (16, 32). Schreiber et al. (16), for example, showed in their prospective quasi-experimental study that opening the doors of acute psychiatric wards is indeed feasible without increasing the risk for critical incidents; but, this study comes along with some methodological issues that have to be kept in mind.

In our study, groups with patients who were first admitted to a ward with an open-door, then transmitted to a ward with a closed-door policy seem to be of particular interest as they have three times higher odds to experience coercive measures. This observation is not astounding. Patients are usually transferred to a closed-doors policy ward because of a critical incident, like aggressive behavior (33). This, in turn, increases the probability for coercive measures (12, 25). Consequently, it seems crucial to address this subgroup in future research, especially when aiming to reduce coercive measures (12, 25, 33). Clinical strategies enabling teams to keep patients on the verge of coercion in treatment in an open-door setting and avoid transfer to a closed ward. This might help to further reduce the incidence of coercion in inpatient psychiatry. However, implementing these strategies requires an adequate patient-staff ratio and well-trained staff (34). Otherwise, one cannot expect with positive effects of opening the doors; on the contrary, there could even be an increase in adverse events like aggression and self-harm.

To the best of the authors’ knowledge, this is the first study including the ward type of the most recent inpatient stay and its association with the use of coercive measures during the current stay. Based on routine data of a large psychiatric university hospital with a health care mandate for psychiatric patients in the canton of Basel-City, potential effects of shifts in patient distribution to other hospitals can be assumed to be negligible. The naturalistic sample with a long observation period and a large number of cases as well as its high external validity are additional strengths.

One limitation of the present study is the inclusion of numerous wards that are at different stages in the opening process. However, this may rather have weakened potential effects as strong effects on certain wards would have been equalized by weaker effects on other wards. Furthermore, the investigation of all wards increases generalizability. Another limitation may be the sizes of the groups. We decided to merge two groups. All other groups were kept in spite of small numbers as we considered especially the groups with patients treated first on a ward with an open-door policy and then transferred to a ward with a closed-door policy during the same stay to be of particular clinical relevance. Third, in our sample, more than 334 patients (6.5%) had more than 11 stays during the observation period. To account for the correlation among the multiple observations in the same patient, we used GEE in our analyses. Thus, the presented estimates are population average effects rather than subject specific estimates. Yet, it would be interesting to investigate the subgroup of high-utilizers of psychiatric inpatient care in future research. Fourth, we did not analyze seclusion and forced medication separately due to the rare occurrence of forced medication without seclusion in our sample. We assume that the driving force behind the association between the sequence of ward types and the use of coercive measures is seclusion based on its frequency in our sample. However, no conclusion can be made about any of both coercive measures individually.

The present study provides additional evidence that open-door policies in psychiatric hospitals are associated with fewer coercive measures, even in patients with previous clinical experience of closed-door policies. The group of patients who were admitted to a ward with an open-door, then transmitted to a ward with a closed-door policy seem to be particular prone to experience coercive measures. Clinical strategies enabling teams to keep patients on the verge of coercion in treatment in an open-door setting might thus have the potential to further reduce the incidence of coercion in inpatient psychiatry.

## Data availability statement

The raw data supporting the conclusions of this article will be made available by the authors, without undue reservation.

## Ethics statement

The studies involving humans were approved by Ethics Committee of Northwestern and Central Switzerland (EKNZ; Project-ID: 287–13 / PB_2020–00029). The studies were conducted in accordance with the local legislation and institutional requirements. Written informed consent for participation was not required from the participants or the participants’ legal guardians/next of kin because obtaining informed consent from all patients who were treated during the observation period of 10 years would have been a disproportionately large effort and also barely possible (e.g., due to relocation or death of patients). The study was therefore categorized and accepted by the ethics committee as further use of routine data without consent according to HRA Art.34/HRO.

## Author contributions

JK: Formal analysis, Investigation, Visualization, Writing – original draft. JM: Conceptualization, Writing – review & editing. LI: Data curation, Writing – review & editing. SS: Data curation, Formal analysis, Investigation, Writing – review & editing. LH: Conceptualization, Writing – review & editing. RL: Formal analysis, Supervision, Writing – review & editing. UL: Conceptualization, Formal analysis, Supervision, Writing – review & editing. CH: Conceptualization, Investigation, Supervision, Writing – original draft.
