# Supplementary material for: The association between the admission to wards with open- vs. closed-door policy and the use of coercive measures
Source: Front Psychiatry. 2023 Oct 25;14:1268727. doi: 10.3389/fpsyt.2023.1268727 (PMC10634515; doi:10.3389/fpsyt.2023.1268727)
Supplement: Supplementary file 1 [file Data_Sheet_1.PDF]

Supplemental Material

Zero-order correlations between predictors, confounders and the outcome variable

	Sequence of ward types	O - O	O - O/C	O - C	O/C - O	O/C - C	C - O	C - O/C	C - C	Age	Gender	Forced medication	Seclusion	Involuntary hospitalization	Aggressive behaviour at admission	Auto-aggressive behaviour at admission	Psychotic symptoms at admission	Length of stay	ICD-10: F0	F1	F2	F3	F4	F5	F6	F7	F8	F9	Other Diagnoses	Number of secondary diagnoses	Coercive measures at 2nd stay (outcome variable)
**Sequence of ward types:**																															
**O - O**		1	-0.13	-0.29	-0.15	-0.12	-0.48	-0.13	-0.64	0.05	0.07	-0.02	-0.20	-0.18	-0.21	-0.07	-0.12	-0.03	-0.02	-0.16	-0.09	0.16	0.10	0.03	0.02	-0.02	0.02	0.05	-0.07	-0.14	-0.17
**O - O/C**			1	-0.01	-0.01	-0.01	-0.02	-0.01	-0.03	-0.02	0.01	0.01	-0.02	-0.02	0.02	0.02	0.02	-0.02	-0.01	-0.01	0.02	-0.01	0.00	0.02	0.02	0.00	0.00	-0.01	0.00	-0.01	0.05
**O - C**				1	-0.02	-0.01	-0.05	-0.01	-0.07	-0.02	0.01	-0.01	-0.03	-0.04	0.05	0.02	0.03	-0.03	0.00	0.01	0.03	-0.01	-0.01	-0.01	0.00	0.02	-0.01	-0.02	0.03	0.01	0.08
**O/C - O**					1	-0.01	-0.03	-0.01	-0.04	-0.03	0.00	0.02	0.06	0.00	0.06	0.03	0.05	0.06	0.00	-0.01	0.04	-0.02	0.00	0.00	0.01	-0.01	0.01	-0.01	0.01	-0.01	-0.01
**O/C - C**						1	-0.02	-0.01	-0.03	-0.01	0.01	0.01	0.11	0.01	0.03	0.03	0.03	0.03	0.00	0.02	0.03	-0.03	-0.01	0.00	0.00	0.01	-0.01	-0.01	0.01	0.02	0.06
**C - O**							1	-0.02	-0.12	-0.02	-0.03	0.01	0.07	0.14	0.12	0.05	0.07	0.06	0.00	0.06	0.05	-0.06	-0.05	-0.01	-0.01	0.01	-0.01	-0.01	0.03	0.05	0.02
**C - O/C**								1	-0.03	-0.02	0.01	0.03	0.08	0.05	0.05	-0.01	0.02	0.01	0.01	0.01	0.04	-0.02	-0.02	0.00	0.00	0.00	-0.01	-0.01	0.01	0.01	0.10
**C - C**									1	-0.02	-0.08	0.01	0.17	0.13	0.13	0.02	0.07	-0.02	0.02	0.16	0.03	-0.13	-0.09	-0.03	-0.03	0.01	-0.01	-0.03	0.05	0.14	0.16
**Age**										1	0.08	0.01	-0.03	0.04	-0.03	-0.14	-0.02	0.20	0.22	-0.03	-0.01	0.13	-0.09	-0.11	-0.24	-0.03	-0.08	-0.17	0.04	-0.12	-0.04
**Gender**											1	0.00	-0.02	0.02	-0.01	0.07	-0.05	-0.04	-0.01	-0.23	-0.04	0.10	0.09	0.13	0.10	0.00	-0.05	-0.09	0.03	-0.13	-0.02
**Forced medication**												1	-0.01	0.10	0.04	-0.01	0.06	0.04	0.00	-0.02	0.06	-0.03	-0.02	0.00	-0.02	0.00	0.01	0.00	-0.01	-0.03	0.02
**Seclusion**													1	0.35	0.24	0.01	0.21	0.11	0.03	-0.03	0.15	-0.09	-0.08	-0.02	-0.04	0.01	-0.01	-0.02	0.02	-0.04	0.27
**Involuntary hospitalization**														1	0.30	0.02	0.22	0.05	0.08	-0.02	0.16	-0.13	-0.09	-0.02	-0.06	-0.01	0.00	-0.02	-0.01	-0.06	0.19
**Aggressive behaviour at admission**															1	0.14	0.25	0.01	0.06	0.00	0.14	-0.14	-0.10	-0.04	0.02	0.06	0.01	0.01	0.01	-0.01	0.16
**Auto-aggressive behaviour at admission**																1	0.02	-0.01	-0.03	-0.04	-0.07	0.05	0.03	0.08	0.16	0.00	0.02	0.02	0.02	0.03	0.00
**Psychotic symptoms at admission**																	1	0.04	0.05	-0.07	0.59	-0.29	-0.19	-0.08	-0.17	-0.01	-0.02	-0.05	-0.01	-0.10	0.16
**Length of stay**																		1	0.10	-0.02	0.03	0.10	-0.07	-0.01	-0.08	-0.04	-0.01	0.01	0.03	-0.01	-0.04
**ICD-10: F0**																			1	-0.08	-0.09	-0.07	-0.08	-0.03	-0.09	0.02	0.00	-0.03	0.05	0.00	0.01
**F1**																				1	-0.09	-0.08	-0.05	-0.01	0.10	-0.05	-0.02	0.08	0.03	0.64	-0.02
**F2**																					1	-0.43	-0.22	-0.09	-0.22	0.01	-0.04	-0.05	-0.01	-0.11	0.15
**F3**																						1	0.01	0.05	0.00	-0.07	0.01	-0.02	0.03	0.10	-0.09
**F4**																							1	0.06	0.09	0.00	0.02	0.03	-0.01	0.16	-0.07
**F5**																								1	0.16	-0.03	-0.01	0.02	0.02	0.12	-0.02
**F6**																									1	-0.01	0.00	0.14	-0.02	0.32	-0.03
**F7**																										1	0.07	0.03	0.07	0.07	0.01
**F8**																											1	0.10	-0.01	0.04	-0.01
**F9**																												1	-0.01	0.20	-0.01
**Other Diagnoses**																													1	0.39	0.00
**Number of secondary diagnoses**																														1	-0.04
**Coercive measures at 2nd stay (outcome variable)**																															1
